# Vibrational Transportation on a Platform Subjected to Sinusoidal Displacement Cycles Employing Dry Friction Control

**DOI:** 10.3390/s21217280

**Published:** 2021-11-01

**Authors:** Sigitas Kilikevičius, Algimantas Fedaravičius

**Affiliations:** Department of Transport Engineering, Kaunas University of Technology, Studentų St. 56, 51424 Kaunas, Lithuania; algimantas.fedaravicius@ktu.lt

**Keywords:** handling and transportation, dry friction, control, vibrations, sinusoidal excitation, motion control

## Abstract

Currently used vibrational transportation methods are usually based on asymmetries of geometric, kinematic, wave, or time types. This paper investigates the vibrational transportation of objects on a platform that is subjected to sinusoidal displacement cycles, employing periodic dynamic dry friction control. This manner of dry friction control creates an asymmetry, which is necessary to move the object. The theoretical investigation on functional capabilities and transportation regimes was carried out using a developed parametric mathematical model, and the control parameters that determine the transportation characteristics such as velocity and direction were defined. To test the functional capabilities of the proposed method, an experimental setup was developed, and experiments were carried out. The results of the presented research indicate that the proposed method ensures smooth control of the transportation velocity in a wide range and allows it to change the direction of motion. Moreover, the proposed method offers other new functional capabilities, such as a capability to move individual objects on the same platform in opposite directions and at different velocities at the same time by imposing different friction control parameters on different regions of the platform or on different objects. In addition, objects can be subjected to translation and rotation at the same time by imposing different friction control parameters on different regions of the platform. The presented research extends the classical theory of vibrational transportation and has a practical value for industries that operate manufacturing systems performing tasks such as handling and transportation, positioning, feeding, sorting, aligning, or assembling.

## 1. Introduction

Objects can be moved using either a nonprehensile or a prehensile method. Prehensile methods usually employ grippers of various designs [1,2]. Nonprehensile methods are preferred in many industrial applications as there is no involvement of gripping, squeezing, picking up, or any other force directly acting on the object [3]. In this case, the objects to be transported are subjected only to unilateral constraints. Due to this, the stress on the object is minimum. This is especially important for the transportation of various fragile or sensitive components. Another important aspect is that the equipment for nonprehensile methods may be more technically feasible compared to grasping equipment and, at the same time, offer better performance. This is especially important for handling and transportation of micro components, as grasping is extremely complicated in this case.

Considerable attention is currently being paid to the problems of motion control, as there are many processes in various manufacturing systems where various objects require handling, transportation, orientation, sorting, classification, or automated assembly. Therefore, this is a very relevant topic in science and modern engineering for a vast range of industries such as automotive, aeronautical, electronic, mechatronics, medicine, or biotechnology. There are many methods to move an object in a nonprehensile way, such as pushing by various robot arms [4,5], catching [6], batting/juggling [7,8,9], employing various planar manipulators [10,11,12,13,14], using various acoustic manipulation systems [15,16,17,18], or using active transportation surfaces where the object’s motion is achieved through controlled deformations generated by arrays of actuators [19]. However, vibration assisted methods, where friction phenomena play a major role, are some of the most versatile, easily implemented, cost effective, and efficient methods [20]. These transportation methods are very versatile because they can be used to transport objects of various sizes and various temperatures, and they are equally suitable for bulk materials and individual objects. Moreover, objects can be transported at long distances, and vibratory transportation equipment can operate in contaminated environments.

A system asymmetry is a necessary condition to achieve the effect of vibrational transportation on an oscillating platform because it ensures that the net friction forces over one displacement cycle of the platform do not cancel out. The dynamics of the object’s motion on a longitudinally vibrating surface were analyzed by Reznik et al. [21]. A time-asymmetry was created through an asymmetric excitation by holding the forward motion for a longer duration than for the backward motion in each oscillation cycle. This time-asymmetry, along with the non-linear nature of friction, resulted in the transportation of the object along a straight-line trajectory. Hunnekens et al. [22] studied self-alignment of an object placed on a vibrating table and showed that a suitable periodic motion profile of the table creates a time-asymmetry, which allows movement of an object along a preferred direction. Moreover, the object can be stopped at a certain position by employing a region of increased friction. The positioning principle is to stop the mass due to an increase in friction when it enters the high-friction region of the table. Experimental validation of this method was done by Hoof et al. [23]. However, these approaches do not provide the ability of dynamic friction control. Mayyas [24,25] analyzed the stick-slip motion dynamics of an object on an oscillating platform suspended by a nonlinear leaf spring. The platform was constructed in such a way that the elastic constant of the spring was direction dependent. A time-asymmetry was created because of the nonlinear properties of the spring, as the forward acceleration of the platform was not equal to the backward acceleration. The study demonstrated that optimum sets of the system natural frequencies and the excitation parameters ensure required velocities at certain dry friction conditions.

Yamaguchi et al. [3,26,27] proposed a method to move objects by employing a vibrating turntable actuated by a single actuator and analyzed the influence of the friction properties of the turntable surface on the velocity map of the object. The motion of objects on the turntable was achieved by controlling the shape and orientation of the geometrically asymmetric orbit of the turntable by changing the offset angle and frequency of the sinusoidal signal for the actuator. Viswarupachari et al. [28] analyzed the transportation of particles on a platform achieved through both a geometric asymmetry and a time-asymmetry, which were induced by asymmetric oscillations.

Blekhman et al. [29] investigated the vibrational transportation of objects along a platform subjected to rotational oscillations. In this case, the transportation was possible due to the kinematic asymmetry created by the rotational excitation. Blekhman et al. [30] analyzed the drive dynamics of vibrational transportation taking place on surfaces subjected to rectilinear oscillations, which cause a kinematic asymmetry. Frei et al. [31] demonstrated that friction forces are capable of transporting objects along a planar horizontal surface by vibrating the surface with two degrees of freedom in order to create a kinematic asymmetry. The method designed by Frei et al. [31] combined the horizontal and vertical oscillations to produce a non-zero resultant friction force; the objects can be moved along any horizontal direction with variable speed.

Umbanhowar et al. [32] analyzed the effects of anisotropic friction of textured surfaces, such as micro-machined silicon and fabrics, on a vibrating platform and showed that direction-dependent surface friction characteristics can be utilized to create friction-induced velocity fields on the surface of the platform for vibratory transportation. Transportation of granular material particles in a vibrating trough with finlike asperities was analyzed by Chen et al. [33]. The directional transportation was possible due to the force asymmetry induced by the finlike asperities.

Another widely known type of asymmetry is a wave asymmetry. This type of asymmetry is induced by traveling waves [34,35,36]. In this case, the object to be transported moves along the direction of the propagation of the travelling wave. Various types of waves can be employed to induce this asymmetry. Kumar and DasGupta [37] used a wave asymmetry for the vibrational transportation of particles by exciting circumferential harmonic traveling waves on a thin circular plate. Minikes and Bucher [38] analyzed the dynamics of the nonprehensile lateral transportation of objects employing a gas squeeze film generated by traveling flexural waves of the driving surface. Zouaghi et al. [39] studied the transport mechanism of dielectric particles in a system with traveling electric field waves.

Reznik et al. [40] excited a plate using several actuators in an asymmetric manner to create average frictional force fields that they called “jets”. By inducing these “jets” at different locations, bodies can be displaced a small amount. Dunst et al. [41] applied the approach of vibration induced friction reduction for handling of dry fine powders. A pipe was used for handling that was subjected to axial low frequency oscillations, and radial high frequency vibrations were employed to manipulate frictional forces. This method showed promising results in increasing the flowability as well as in decreasing adhesion and agglomeration. Kilikevičius et al. [42] employed a platform subjected to circular motion for nonprehensile omnidirectional manipulation tasks, and dynamic dry friction was dynamically controlled to create a frictional system asymmetry. A similar approach was applied for the manipulation of miniature and microminiature bodies [43].

In summary, an asymmetry is a necessary condition to achieve the motion of an object placed on an oscillating platform. Well-known asymmetries that are currently most widely applied for vibrational transportation can be classified into the following groups: geometric or spatial asymmetry (when the system forms an angle with the horizontal plane), kinematic asymmetry (when the direction of the excited oscillations forms an angle with the movement direction), wave asymmetry (where the object to be transported moves along the direction of the propagation of the travelling wave), and time-asymmetry (when the forward motion and the reverse motion of the platform have different velocities). This paper presents a new method of the vibrational transportation of objects on a platform, subjected to sinusoidal displacement cycles, achieved through dynamic dry friction control. Hereby, a preferred directional motion is maintained through the asymmetry of frictional conditions, which is created by periodic dynamic control of the frictional force between the object and the platform with respect to the period of the sinusoidal excitation of the platform. It is technically feasible through various means, e.g., the effective time-averaged dry friction force can be dynamically reduced by exciting high-frequency vibrations in the contact zone between the object to be transported and the platform. It was demonstrated by numerous scientists [44,45,46,47,48,49,50] that high-frequency vibrations cause a reduction in the effective time-averaged friction force. The friction force can also be controlled by periodically increasing the normal force by using pneumatic suction forces. If the object is made of ferromagnetic materials, magnetic forces can be used as well to increase the normal force periodically. The objectives of the presented research are to theoretically and experimentally verify that vibrational transportation can be achieved through dynamic dry friction control, to reveal the functional capabilities of the proposed vibrational transportation method, and to identify the control parameters that determine the transportation characteristics such as velocity and direction.

## 2. Theoretical Research

### 2.1. Mathematical Model

**Figure 1:**
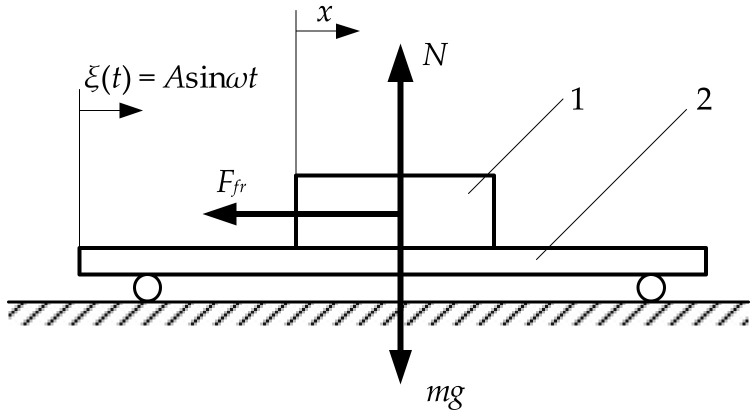
Lumped-element model of vibrational transportation: (1) object to be transported; (2) platform subjected to sinusoidal displacement cycles.

Figure 1 presents the lumped-element model of the vibrational transportation of an object on a horizontal platform, which is achieved through dynamic dry friction control.

The platform is subjected to sinusoidal displacement cycles in the longitudinal direction:

(1) $$\xi \left(t\right)=A\sin\omega t,$$

where *t* is time, *A* is the amplitude of the sinusoidal excitation, and *ω* is the angular frequency of the sinusoidal excitation.

The equation of the local object motion on the platform is written as follows:

(2) $$m\ddot{x}=mA\omega^{2}\sin\omega t-F_{fr},$$

where *m* is the mass of the object, *x* is the local displacement of the object, and *F_fr_* is the dry friction force between the surfaces of the object and the platform.

**Figure 2:**
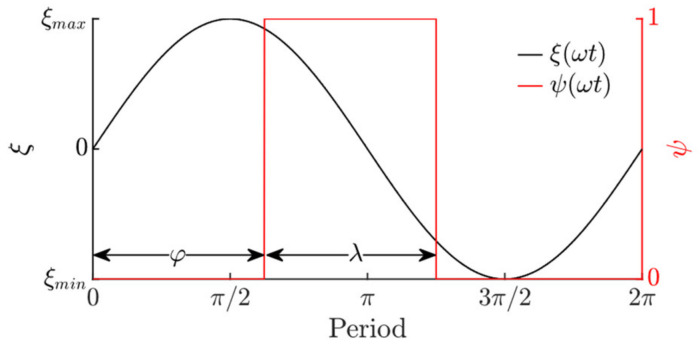
Duty cycle function *ψ*(*τ*) for dry friction control with respect to the period of sinusoidal displacement cycles.

In order to ensure that net friction forces over one sinusoidal displacement cycle do not cancel out, this paper proposes a new type of asymmetry of frictional conditions, which is achieved through dynamic dry friction control. In this case, the dry friction force, which is controlled in respect of the period of the sinusoidal excitation, is expressed as follows:

(3) $$\left\{\begin{array}{c} F_{fr}=mg\mu \left(\psi \right)\mathrm{sign}\dot{x},\;\mathrm{if}\;\dot{x}\neq 0,\\ -mg\mu \left(\psi \right)<F_{fr}<mg\mu \left(\psi \right),\;\mathrm{if}\;\dot{x}=0, \end{array}\right.$$

where *g* is the gravitational acceleration (in this study it was assumed to be 9.81 m/s^2^), and *μ*(*ψ*) is the kinetic friction coefficient, which is controlled by a duty cycle control signal. In this case, the duty cycle is determined as follows:

(4) $$\psi \left(\omega t\right)=\left\{\begin{array}{c} 1,\;\mathrm{if}\;\varphi +2\pi n<\tau <\varphi +\lambda +2\pi n,\\ 0,\;\mathrm{otherwise}, \end{array}\right.$$

where *n* is the cycle number, *φ* is the phase shift between the pulse function for dynamic dry friction control and the sinusoidal excitation, and *λ* is the pulse width of the duty cycle (Figure 2).

In practice, the effective time-averaged dry friction force can be controlled by installing functional elements for friction control in the platform. For example, piezoelectric actuators can be installed in the platform to periodically decrease the effective dry friction coefficient by exciting high frequency vibrations. When the elements for friction control are activated (ψ=1), the time-averaged effective value of the friction coefficient is dynamically changed ($\mu \left(1\right)={\bar{\mu }}_{m}$), and, when the elements for friction control are deactivated (ψ=0), the effective friction coefficient has its nominal value ($\mu \left(0\right)=\mu_{0}$). Such periodic dynamic dry friction control causes an asymmetry of frictional conditions, which is necessary to make the object move.

### 2.2. Modeling Results

To investigate the functional capabilities of the proposed method and to identify the control parameters that determine the transportation characteristics such as velocity and direction, a numerical modeling of the vibratory transportation on a platform employing dynamic dry friction control was carried out. The software for the modeling was developed using the MATLAB (MathWorks, Natick, MA, USA) programing language. The mathematical model was numerically solved using the Runge-Kutta ordinary differential equation solver ode45s with an adaptive time-stepping scheme.

**Figure 3:**
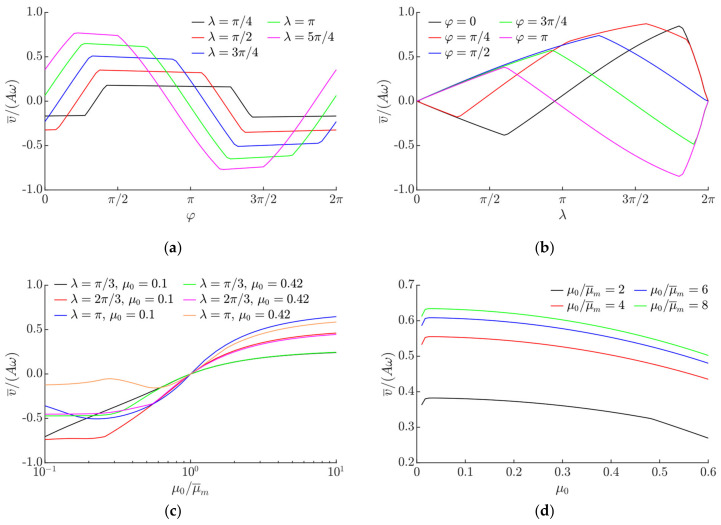
Normalized average transportation velocity depending on: (**a**) *φ* when *A* = 1 mm, *ω* = 125.66 rad/s, $\mu_{0}$ = 0.1, $\mu_{0}/{\bar{\mu }}_{m}$ = 8; (**b**) *λ* when *A* = 1 mm, *ω* = 125.66 rad/s, $\mu_{0}$ = 0.1, $\mu_{0}/{\bar{\mu }}_{m}$ = 8; (**c**) $\mu_{0}/{\bar{\mu }}_{m}$ when *A* = 1 mm, *ω* = 125.66 rad/s, *φ = π*/2; (**d**) $\mu_{0}$ when *A* = 1 mm, *ω* = 125.66 rad/s, *λ = π*, *φ = π*/2.

Figure 3 demonstrates the modeling results of the normalized average transportation velocity $\bar{v}/\left(A\omega \right)$. The modeling results clearly demonstrate that the direction and velocity of the object can be easily controlled in a wide range by changing the parameters of the duty cycle controlling the effective dry friction coefficient.

As *φ* increases, the average transportation velocity tends to increase as well, and, depending on *λ*, reaches its maximum value when *φ* is approximately *π*/2 rad. Under further increase in *φ*, the average transportation velocity does not change much and maintains a value near its maximum. However, at some point, the average transportation velocity starts to decrease again and ultimately the direction of the motion is reversed. The range of *φ*, where the average transportation velocity maintains a value near its maximum, depends on *λ*. Under lower values of *λ*, the direction of the motion is reversed at higher values of *φ*. The maximum value of the average transportation velocity towards the negative direction is observed when *φ* is approximately 3*π*/2. Parameter combinations for the control of the transportation velocity are presented in Figure 3a. Judging from the nature of the dependencies of $\bar{v}/\left(A\omega \right)$ on the phase shift *φ* (Figure 3a), this control parameter (*φ*) is the most suitable to control the direction of transportation. The nature of the influence of *φ* on the transportation velocity can be explained by the fact that this parameter defines the shift between the duty cycles, which causes the asymmetry of frictional properties and the period of the sinusoidal excitation.

When *λ* increases, the magnitude of the average transportation velocity increases as well, as higher values of *λ* result in an increase in the asymmetry of frictional conditions. However, when a critical value of *λ* is reached, a further increase in this parameter results in a decrease in the magnitude of the average transportation velocity (Figure 3b). This is related to a decrease in the asymmetry of frictional conditions when this critical value is exceeded. The critical value depends on *φ*, as this parameter defines the shift between the duty cycles, which causes the asymmetry of frictional properties and the period of the sinusoidal excitation.

**Figure 4:**
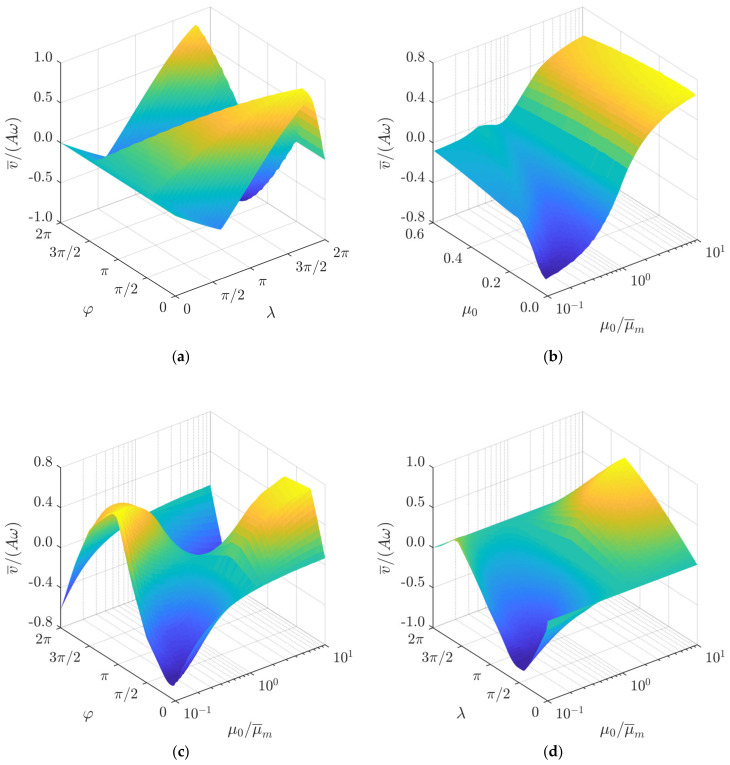
Normalized average transportation velocity depending on: (**a**) *φ* and *λ* when *A* = 1 mm, *ω* = 125.66 rad/s, $\mu_{0}$ = 0.1, $\mu_{0}/{\bar{\mu }}_{m}\;$ = 8; (**b**) *μ_c_*_1_ and $\mu_{0}/{\bar{\mu }}_{m}$ when *A* = 1 mm, *ω* = 125.66 rad/s, *λ = π*, *φ = π*/2; (**c**) *φ* and $\mu_{0}/{\bar{\mu }}_{m}$ when *A* = 1 mm, *ω* = 125.66 rad/s, *λ = π*, $\mu_{0}$ = 0.1; (**d**) *λ* and $\mu_{0}/{\bar{\mu }}_{m}$ when *A* = 1 mm, *ω* = 125.66 rad/s, $\mu_{0}$ = 0.1 *φ = π*/2.

Another important control parameter is the rate of the dynamic modification of the effective dry friction coefficient, which is expressed as $\mu_{0}/{\bar{\mu }}_{m}$. This parameter also determines the magnitude of the asymmetry of frictional conditions. The influence of $\mu_{0}/{\bar{\mu }}_{m}$ on the normalized average transportation velocity is presented in Figure 3c. The modeling results demonstrate that in order to achieve a directional motion, the dry friction force can be controlled by either periodically increasing or periodically decreasing the effective friction coefficient, i.e., modifying it in both directions from the nominal value. Values of $\mu_{0}/{\bar{\mu }}_{m}$ further from 1 result in higher values of the average transportation velocity. At lower values of $\mu_{0}$, higher values of the average transportation velocity were observed when dry friction was periodically being increased. In contrast, at higher values of $\mu_{0}$, higher values of the average transportation velocity were observed when dry friction was periodically being decreased. This is due to the fact that at higher values of $\mu_{0}$, a higher asymmetry was created when dry friction was periodically being decreased, whereas, at lower values of $\mu_{0}$, a higher asymmetry was created when dry friction was periodically being increased. Figure 3d shows the influence of $\mu_{0}$ on the average transportation velocity at values of $\mu_{0}/{\bar{\mu }}_{m}$ that are higher than 1. In this case, the average transportation velocity tends to decrease if $\mu_{0}$ increases, with an exception for extremely low $\mu_{0}$ values ($\mu_{0}$ < 0.017) (Figure 3d). The response of the normalized average transportation velocity to $\mu_{0}$ and $\mu_{0}/{\bar{\mu }}_{m}$ is demonstrated in Figure 4b. It also shows that when $\mu_{0}/{\bar{\mu }}_{m}$ < 1, an increase in $\mu_{0}$ results in a significant decrease in the absolute normalized average velocity in a range of $\mu_{0}$ up to approximately 0.2. A further increase in $\mu_{0}$ does not have such a significant influence on the normalized transportation velocity. This behavior is more pronounced at values of $\mu_{0}/{\bar{\mu }}_{m}$ that are closer to 10^−1^ (Figure 4b). The combined influence of *φ* and $\mu_{0}/{\bar{\mu }}_{m}$ on the normalized average transportation velocity is presented in Figure 4c, and the combined influence of *λ* and $\mu_{0}/{\bar{\mu }}_{m}$ on the normalized average transportation velocity is shown in Figure 4d.

**Figure 5:**
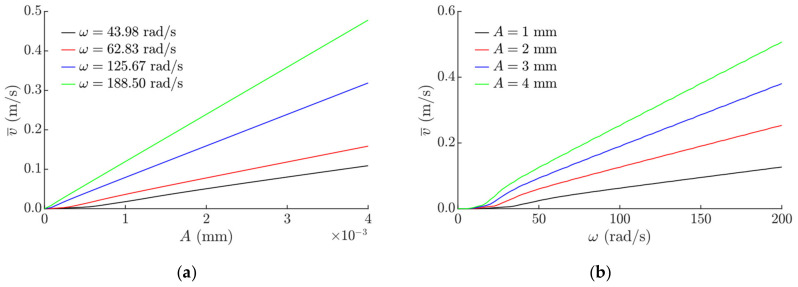
Average transportation velocity depending on: (**a**) *A* when $\mu_{0}$ = 0.1, $\mu_{0}/{\bar{\mu }}_{m}\;$ = 8, *λ = π*, *φ = π*/2; (**b**) *ω* when $\mu_{0}$ = 0.1, $\mu_{0}/{\bar{\mu }}_{m}\;$ = 8, *λ = π*, *φ = π*/2.

The average transportation velocity depending on the amplitude of the sinusoidal excitation is presented in Figure 5. An increase in the amplitude results in an increase in the average transportation velocity as well (Figure 5a). A linear dependance between the average velocity and the amplitude was observed. Only at very small amplitudes and frequencies were some nonlinearities present. Similarly, a nonlinear dependence between the average velocity and the angular frequency was observed in a range up to 40 rad/s. At higher frequencies, the transportation velocity linearly increases when the angular frequency increases (Figure 5b).

**Figure 6:**
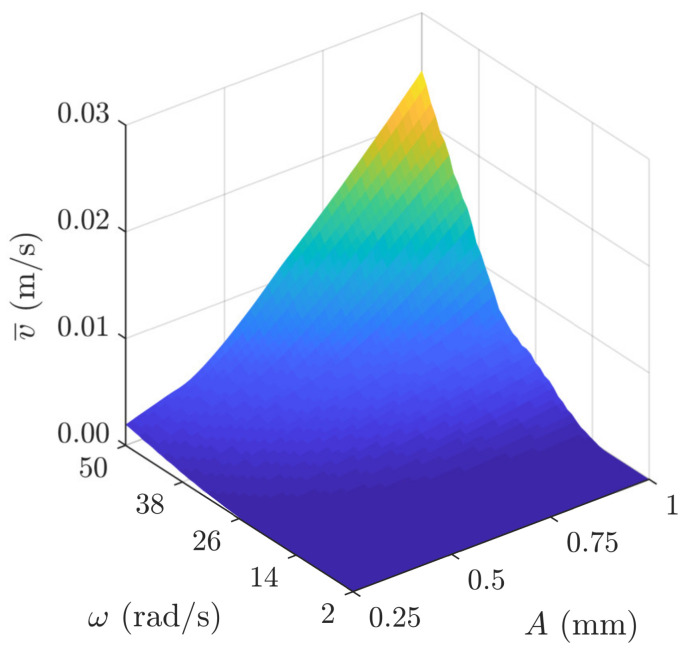
Three-dimensional representation of the average transportation velocity as a function of the amplitude and the angular frequency of the sinusoidal excitation in a range of low *A* and *ω* values.

Figure 6 shows the three-dimensional representation of the average transportation velocity as a function of the amplitude and the angular frequency of the sinusoidal excitation in a range of low *A* and *ω* values.

## 3. Experimental Research

### 3.1. Experimental Setup and Methodology

**Figure 7:**
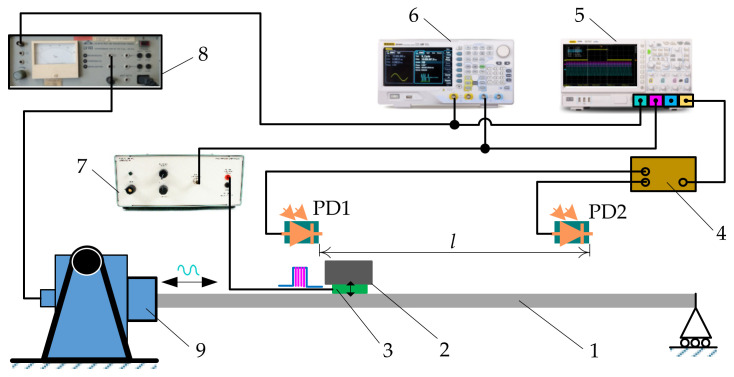
Schematic diagram of the experimental setup used to measure the average transportation velocity: (1) platform; (2) part to be transported; (3) piezoelectric actuator; (4) signal generator for photodiode sensors (PD1 and PD2); (5) digital oscilloscope; (6) arbitrary waveform generator; (7) high-frequency vibration amplifier; (8) power amplifier for the sinusoidal excitation; (9) electrodynamic shaker.

An experimental investigation was carried out in order to test the functional capabilities of the proposed method. Figure 7 presents the schematic diagram of the experimental setup, which was used for the measurements of the average transportation velocity.

**Figure 8:**
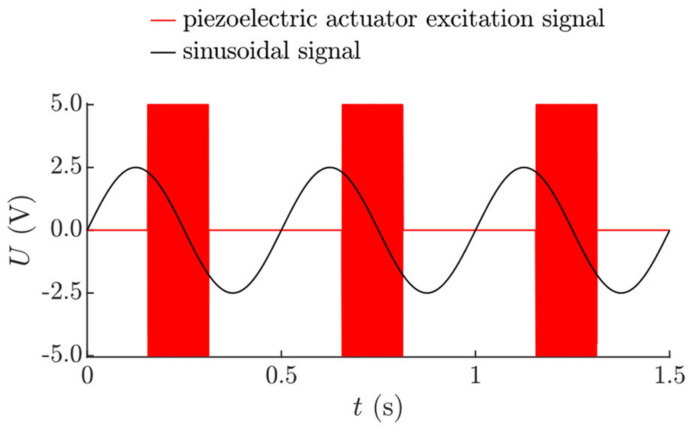
Oscillograms of the sinusoidal signal and the signal for the piezoelectric actuator excitation as *ω* = 125.66 rad/s, *λ* = 3*π*/8, and *φ* = 5*π*/8.

The platform was subjected to sinusoidal displacement cycles in the longitudinal direction using an ESE 211 electrodynamic shaker (VEB Robotron-Meßelektronik, Dresden, Germany). In this research, a piezoelectric actuator was used to excite high-frequency vibrations of 25 kHz in the contact zone. In practical implementations of this vibrational transportation method, piezoelectric actuators can be installed in the platform. However, in this experimental setup, the piezoelectric actuator was placed under the part to be transported for easier manufacturing, since the objectives of the experiments were to verify that vibrational transportation can be achieved through dynamic dry friction control, to test functional capabilities observed in the theoretical research, and to identify the control parameters that determine the transportation characteristics. When the piezoelectric actuator excites high-frequency vibrations, the dynamic processes that take place in the contact zone result in a reduction in the effective time-averaged friction force. This allows for the dynamic control of the dry friction force between the part and the platform in a predefined way. The parameters of the sinusoidal excitation and the high-frequency excitation were controlled by a DG4202 arbitrary waveform generator (RIGOL, Beijing, China). The high frequency vibrations of the piezoelectric actuator were being periodically turned on and off by a duty cycle control signal, which was coordinated with respect of the period of the sinusoidal excitation in the same manner as shown in Figure 2 (the duty cycle signal was synchronized with the sinusoidal excitation signal by the arbitrary waveform generator). In such a way, the piezoelectric actuator was being activated during each period of the sinusoidal excitation for a fraction of the period equal to *λ* with a phase shift of *φ*. This control manner is illustrated by the voltage *U* oscillograms of the sinusoidal signal and the signal for the piezoelectric actuator excitation (Figure 8).

The harmonic excitation signal was amplified by an LV-103 power amplifier (Metra Mess- und Frequenztechnik in Radebeul, Radebeul, Germany) and fed to the electrodynamic shaker, and the signal for the piezoelectric actuator was amplified by an EPA-104 piezo linear amplifier (Piezo Systems Inc., Cambridge, MA, USA). Photodiode sensors PD1 and PD2 were used to register the time taken for the part to be transported between the two points separated by a distance of *l* = 0.2 m. When the moving part was crossing the photodiode sensors (PD1 and PD2), the corresponding signals were generated at these exact time instances. The signals from the photodiode sensors and the arbitrary waveform generator were monitored by a DS1054 digital oscilloscope (RIGOL, Beijing, China). In this way, the time *t_tr_* taken for the object to be transported between the photodiode sensors was measured using the digital oscilloscope. The experimental average transportation velocity $\bar{v}$ was calculated by dividing the covered distance by this time *t_tr_*.

### 3.2. Experimental Results and Comparison with the Model

**Figure 9:**
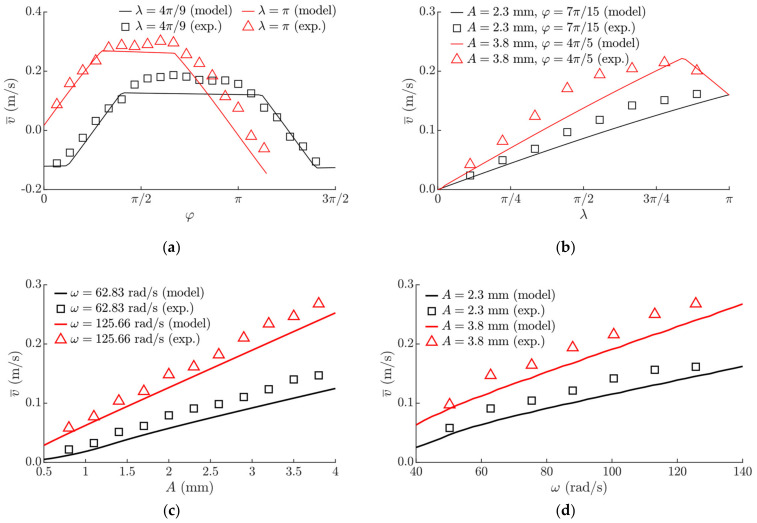
Experimental and modeled dependences of the average transportation velocity depending on: (**a**) *φ* when *A* = 3.8 mm, *ω* = 125.66 rad/s, $\mu_{0}$ = 0.24, $\mu_{0}/{\bar{\mu }}_{m}\;$ = 4; (**b**) *λ* when *ω* = 125.66 rad/s, $\mu_{0}$ = 0.24, $\mu_{0}/{\bar{\mu }}_{m}$ = 4; (**c**) *A* when $\mu_{0}$ = 0.24, $\mu_{0}/{\bar{\mu }}_{m}\;$ = 4, *λ =* 8*π*/9, *φ =* 7*π*/15; (**d**) *ω* when $\mu_{0}$ = 0.24, $\mu_{0}/{\bar{\mu }}_{m}\;$ = 4, *λ =* 8*π*/9, *φ =* 7*π*/15.

Experiments of vibrational transportation employing dry friction control were caried out using the experimental setup presented in Figure 7. The high-frequency excitation reduced the effective dry friction force between the sliding surfaces of the experimental setup up to four times, and the nominal friction coefficient was 0.24. The frictional properties of the experimental setup were determined by using the horizontal surface method. The experiments have demonstrated that the proposed vibrational transportation method is feasible from a technical point of view and can be applied in practice. The part was moving on the platform, and the transportation characteristics depended on the control parameters that were determined in the theoretical research. Figure 9 demonstrates the results of velocity measurements. The experimental investigation confirmed the theoretical observation that the transportation direction can be controlled by manipulating the phase shift *φ*. The influence of the phase shift *φ* on the average transportation velocity is shown in Figure 9a. The influence of *λ* on $\bar{v}$ is presented in Figure 9b. It shows that the average velocity can be smoothly controlled by adjusting *λ*. This was consistent with the theoretical findings. The influence of the sinusoidal excitation parameters on the average transportation velocity was experimentally determined. It showed that an increase either in the amplitude of the sinusoidal excitation (Figure 9c) or in the angular frequency (Figure 9d) results in an increase in the average transportation velocity.

The obtained experimental results were compared with the modeling results (Figure 9). The experimental values of the average transportation were observed to be somewhat higher than the values obtained by the modeling. One of the reasons of this might be because the dependence of the effective friction coefficient on the relative sliding velocity was not considered in the model. However, the differences were within an acceptable range, and the average transportation velocity observed by the experimental measurements followed the same trends as estimated by the modeling.

## 4. Discussion

The results of the presented research indicate that the method of vibrational transportation, achieved through dynamic dry friction control, ensures smooth control of the transportation velocity in a wide range and allows for changing the direction of motion. This is an advantage over other methods such as vibrational conveyors employing kinematic asymmetries when the direction of the excited oscillations forms an angle with the movement direction. In this a case, the orientation of such a device would be needed to be reversed in order to change the transportation direction. Moreover, the proposed method offers other new functional capabilities such as a capability to move individual objects on the same platform in opposite directions and at different velocities at the same time. This can be achieved by installing elements for dynamic dry friction control on different regions of the platform and imposing different friction control parameters on these separate regions. It also can be done by imposing different friction control parameters on different objects to be transported. This capability is demonstrated by an experiment presented in Supplementary Material Video S1. In addition, an object on the platform can be subjected to translation and rotation at the same time by placing it on a platform that has two separate parallel regions with different nominal friction coefficients. In this case, the object moving forward would also rotate due to the moment induced by the different magnitudes of the net friction forces created on these two separate regions through dynamic dry friction control. This capability is demonstrated by an experiment presented in Supplementary Material Video S2. In addition, objects on the platform can be subjected to translation and rotation at the same time by installing elements for dynamic dry friction control on two parallel separate regions of the platform and imposing different friction control parameters on these two parallel regions. These functional capabilities expand the scope of applications of vibrational transportation as the objects to be transported can also be aligned. These additional functional capabilities and their potential practical applications are subjects for future research.

## 5. Conclusions

The vibrational transportation of objects on a platform subjected to sinusoidal displacement cycles was analyzed by employing dynamic dry friction control.

A parametric mathematical model was developed for the theoretical research on the functional capabilities and transportation regimes of the proposed vibrational transportation method. The modeling verified that a preferred directional motion can be maintained through the asymmetry of frictional conditions, which is created by periodic dynamic control of the frictional force between the object and the platform with respect to the period of the sinusoidal excitation of the platform. The modeling showed that in order to achieve a directional motion, dry friction can be controlled by either periodically increasing or periodically decreasing the effective friction coefficient, i.e., dynamically modifying it in both directions from the nominal value. The modeling identified the control parameters that determine the transportation characteristics. The results clearly demonstrated that the direction and velocity of the object can be easily controlled in a wide range by changing the parameters of the duty cycle controlling dry friction. The phase shift *φ* between the sinusoidal function and the duty cycle is the most suitable parameter to change the transportation direction. The transportation velocity is controlled by adjusting the pulse width *λ* of the duty cycle controlling dry friction, as an increase in this parameter results in an increase in the asymmetry of frictional conditions. As the ratio $\mu_{0}/{\bar{\mu }}_{m}$ also determines the magnitude of the asymmetry of frictional conditions, values of $\mu_{0}/{\bar{\mu }}_{m}$ further from 1 result in higher values of the average transportation velocity. The modeling also showed that an increase in either the amplitude of the sinusoidal excitation or the angular frequency results in an increase in the average transportation velocity as well.

To test the functional capabilities of the proposed method, an experimental setup was developed, and vibrational transportation experiments were carried out. The experiments have demonstrated that the proposed vibrational transportation method is feasible from a technical point of view and can be applied in practice. The influence of the control parameters as well as the sinusoidal excitation parameters on the average transportation velocity was experimentally determined. The experimental investigation confirmed the theoretical observation that the transportation direction can be controlled by manipulating the phase shift *φ*. The transportation velocity was experimentally controlled by adjusting the pulse width *λ* of the duty cycle controlling dry friction. This was also consistent with the theoretical findings. A comparison between the theoretical and experimental results showed that the average transportation velocity observed by the experimental measurements follows the same trends as estimated by the modeling.

The results of the presented research indicate that the method of vibrational transportation achieved through dynamic dry friction control ensures smooth control of the transportation velocity in a wide range and allows one to change the direction of motion. Moreover, the proposed method offers other new functional capabilities, such as a capability to move individual objects on the same platform in opposite directions and at different velocities at the same time by imposing different friction control parameters on different regions of the platform or on different bodies. In addition, the objects on the platform can be subjected to translation and rotation at the same time by imposing different friction control parameters on different regions of the platform. These functional capabilities expand the scope of applications of vibrational transportation as the objects to be transported can also be aligned.

The presented research extends the classical theory of vibrational transportation and has a practical value for industries that operate manufacturing systems performing tasks such as handling and transportation, positioning, feeding, sorting, aligning, or assembling.

## Supplementary Materials

The following are available online at https://www.mdpi.com/article/10.3390/s21217280/s1, Video S1: an experiment of the vibrational transportation of objects on a platform employing dynamic dry friction control; Video S2: an object subjected to translation and rotation at the same time by placing it on the platform, which has two separate parallel regions with different nominal friction coefficients.
